# A Comprehensive Virulence and Resistance Characteristics of *Listeria monocytogenes* Isolated from Fish and the Fish Industry Environment

**DOI:** 10.3390/ijms24043581

**Published:** 2023-02-10

**Authors:** Arkadiusz Józef Zakrzewski, Monika Kurpas, Anna Zadernowska, Wioleta Chajęcka-Wierzchowska, Maria João Fraqueza

**Affiliations:** 1 Department of Industrial and Food Microbiology, University of Warmia and Mazrui, 10-726 Olsztyn, Poland; 2 Department of Immunobiology and Environmental Microbiology, Medical University of Gdansk, 80-210 Gdansk, Poland; 3 CIISA—Centro de Investigação Interdisciplinar em Sanidade Animal, Faculdade de Medicina Veterinária, Universidade de Lisboa, 1749-016 Lisboa, Portugal

**Keywords:** *Listeria monocytogenes*, virulence, WGS, fish

## Abstract

*Listeria monocytogenes* is an important pathogen, often associated with fish, that can adapt and survive in products and food processing plants, where it can persist for many years. It is a species characterized by diverse genotypic and phenotypic characteristics. Therefore, in this study, a total of 17 *L. monocytogenes* strains from fish and fish-processing environments in Poland were characterized for their relatedness, virulence profiles, and resistance genes. The Core Genome Multilocus Sequence Typing (cgMLST) analysis revealed that the most frequent serogroups were IIa and IIb; sequence types (ST) were ST6 and ST121; and clonal complexes (CC) were CC6 and CC121. Core genome multilocus sequence typing (cgMLST) analysis was applied to compare the present isolates with the publicly available genomes of *L. monocytogenes* strains recovered in Europe from humans with listeriosis. Despite differential genotypic subtypes, most strains had similar antimicrobial resistance profiles; however, some of genes were located on mobile genetic elements that could be transferred to commensal or pathogenic bacteria. The results of this study showed that molecular clones of tested strains were characteristic for *L. monocytogenes* isolated from similar sources. Nevertheless, it is worth emphasizing that they could present a major public health risk due to their close relation with strains isolated from human listeriosis.

## 1. Introduction

*Listeria monocytogenes,* as an environmental foodborne pathogen, is considered one of the most significant contaminants in food processing and raw agricultural supplies. *L. monocytogenes* contamination leads to a significant threat for risk groups, including the elderly, pregnant women, neonates, and the immunocompromised [1], for whom fatality rate can reach 20–30% [2]. In recent years, it has been responsible for large-scale outbreaks related to fresh produce, deli meats, and other RTE products worldwide [3]. While listeriosis is often associated with the consumption of contaminated RTE food products, such as cheese, meat and fish products, graved and smoked fish are the most frequently contaminated with *L. monocytogenes* around the world [4,5,6,7,8]. Salmon and salmon products in particular are regarded as important sources of human exposure to *L. monocytogenes* in other countries [9,10].

*L. monocytogenes* cause flu-like symptoms in most patients. However, in the group with risk (elderly, immunocompromised, and pregnant patients), the pathogen is responsible for central nervous system and fetal–placental infection [11]. *L. monocytogenes* can pass important barriers in a host, namely the intestinal epithelium, the blood–brain barrier, and the placenta, and subsequently spread to other organs [12]. The process of Listeria’s infection involves several different stages: adhesion and invasion of host cells, mentioned above, internalization by host cells, lysis of the vacuole with a high division rate comparable to that in rich broth medium, recruitment and polymerization that generate a network of branched filaments, intracellular multiplication, and intercellular spread to the adjacent cell [13]. *L. monocytogenes* is an extremely dangerous and deadly pathogen, due to the numerous mechanisms that allow it to penetrate host cells and survive in them [14].

**Figure 1:**
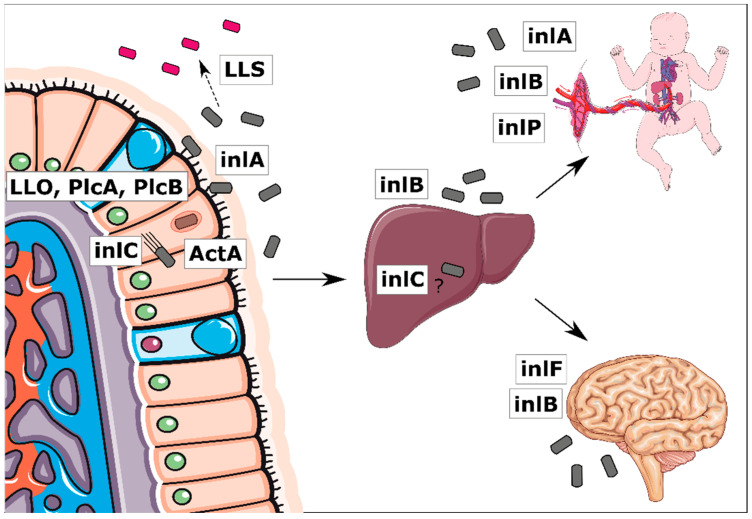
Scheme of *L. monocytogenes* infection including the studied virulence factors [11,14].

The main genetic factors determining the ability to induce listeriosis infection include internalin encoded by the inlAB operon and pathogenic islands (LIPI-1, LIPI-3, and LIPI-4). Both LIPI-1 and the inlAB operon are crucial, and genes clustered there encode adhesion, internalization, intracellular survival, and dissemination [15]. Moreover, internalin A is a major factor inducing the internalization of L. monocytogenes to epithelial cells. Internalin B is important for the placental invasion [11]. LIPI-3 is composed of eight genes that encode listeriolysin S (LLS), a hemolytic and cytotoxic factor important in murine infection, and it participates in polymorphonuclear-cell survival [16,17]. LIPI-4 is a cluster containing six genes encoding a sugar transport system involved in neural and placental infection [18]. A schematic infection model is shown in Figure 1.

*L. monocytogenes* is a heterogenous group of bacteria. This pathogen can be differentiated into 14 different serogroups, which are prevalent and characterized by PCR or MLST (multilocus sequence type). However, since those methods are based on just several genes, they have low discriminatory power. The PFGE method, a gold standard, is able to discriminate different strains but is laborious and requires close and continuous work to avoid errors. At present, due to whole genome sequencing (WGS), the characterization of core genome MLST (cgMLST) is the most suitable method [19]. Since WGS provides the highest discriminatory power, it provides the possibility of identifying the presence of virulence genes, characterizing them, and correlating them with a reported disease severity.

Therefore, the aim of this study was to perform a WGS-based characterization of the genetic difference in *L. monocytogenes* strains isolated from fish and fish-processing environments.

## 2. Results

### 2.1. MLST Analysis

**Figure 2:**
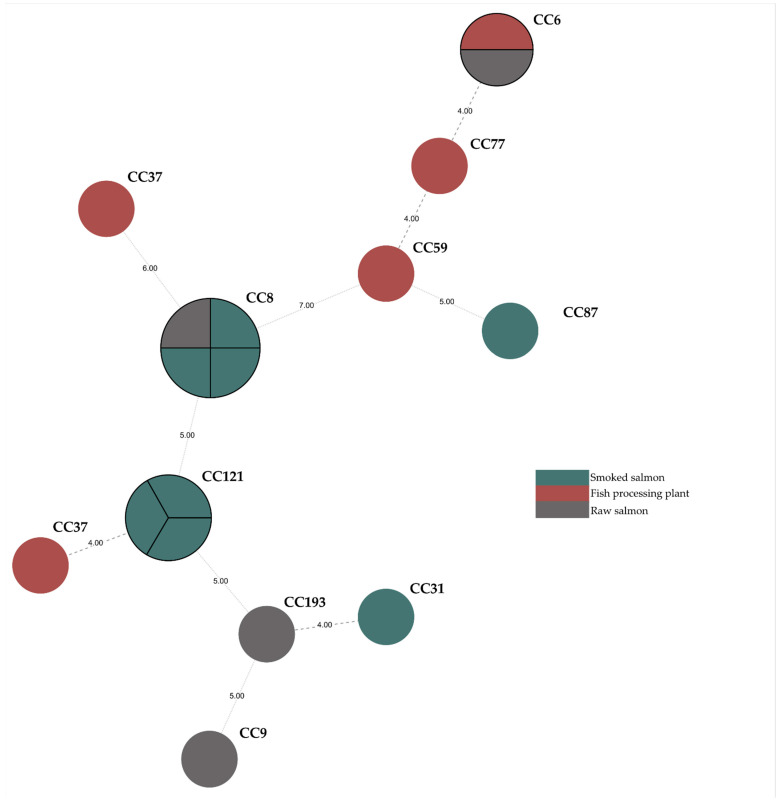
Minimum spanning tree analysis based on the MLST allelic profiles of 17 *L. monocytogenes* isolates. Each circle represents a sequence type (ST). The numbers on the connecting lines illustrate the numbers of target genes with different alleles. The sources of isolates are distinguished by the colors.

Eleven different MLST sequence types (ST) were identified: ST6 (11.76%, *n* = 2 isolates), ST8 (23.53%, *n* = 4), ST9 (5.88%, *n* = 1), ST31 (5.88%, *n* = 1), ST37 (5.88%, *n* = 1), ST59 (5.88%, *n* = 1), ST77 (5.88%, *n* = 1), ST87 (5.88%, *n* = 1), ST101 (5.88%, n = 1), ST121 (17.65%, *n* = 3), and ST193 (5.88%, *n* = 1). *L. monocytogenes* collection from food-premise environments contained isolates classified into six sequence types (ST101, ST69, ST77, ST 37, ST121, and ST87) whereas those from food samples were classified into seven other STs (ST6, ST8, ST9, ST31, ST87, ST121, and ST193). Moreover, *L. monocytogenes* were grouped into eleven clonal complexes (CC6, CC8, CC9, CC31, CC37, CC59, CC77, CC87, CC101, CC121, and CC193) (Figure 2). The obtained results allowed us to determine that the dominant number of strains fit to Lineage II (72.2%) and the remaining strains to Lineage I (27.8%).

### 2.2. cgMLST Analysis

Obtained results showed that tested isolates belonged to 14 different core genome MLST types (CT), with the most (17.65%) belonging to the CT-1151 type, and all these strains were isolated from smoked salmon. Additionally, two strains (11.76%) belonged to the CT909 type and were also isolated from smoked salmon. The occurrence of one strain with CT11711, CT11716, CT11717, CT11718, CT1824, CT2283, CT443, CT5641, CT58, CT7220, CT7227, and CT893 (Figure 2) was observed.

### 2.3. Assessment of Virulence Factor Genotypes across Different Sublineages

The presence and integrity of *Listeria monocytogenes* pathogenicity islands 1 to 4 (LIPI-1 to LIPI-4) and the other 63 genes were investigated. All of the tested strains had genes that belonged to LIPI-1 (six genes: *prfA*, *plcA*, *hly*, *mpl*, *actA*, *and plcB*), twelve strains (70.59%) had only one gene, namely *inl*I that belonged to LIPI-2, and the rest of the genes were absent. The occurrence of the LIPI-3 (eight genes: llsA, llsG, llsH, *llsX*, *llsB*, *llsY*, *llsD*, and *llsP*) was observed in three strains, two from the ST6 serogroup IVb and one from the ST77 serogroup IIb (isolate LM8, isolate LM10, and isolate LM13) (17.65%), while the LIPI-4 island was found in one strain from ST 87 and serogroup IIb (5.88%), isolated from smoked salmon. Based on Genome Comparator, it was found that the tested strains represented 14 unique types, based on virulence profile, and only the Lm_1, Lm_2, Lm_13 and Lm_16 strains belonged to one type.

**Figure 3:**
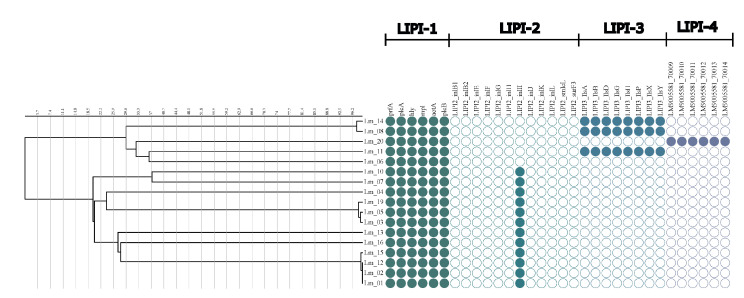
Dendrogram constructed with cgMLST data for the 17 *L. monocytogenes* isolates with gene content characteristics.

Only four genes (*inlA*, *inlE*, *inlH,* and *inlC2*) from the internalin gene family members were found in all isolates tested, whereas the *inlB*, *inlC*, *inlJ*, *inlK,* and *inlD* genes were present on 94.12% of isolates. Moreover, the analysis showed that three of the tested isolates (17.65%) harbored a premature stop codon (PMSC). Isolates with *inlA* gene truncation belonged to serogroup IIa, ST121, ST193, and ST31 (Figure 3).

### 2.4. Antimicrobial Resistance and Stress Tolerance Genes

**Figure 4:**
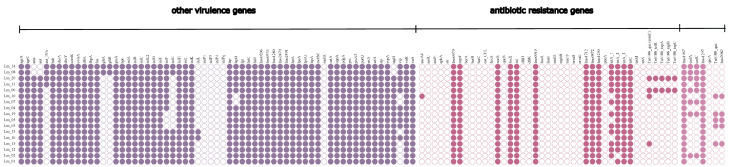
Occurrence of virulence and resistance among *L. monocytogenes* isolates.

The most prevalent antimicrobial resistance factors were those coding tetracycline resistance, including the *lmo0839*, *tet*A_3, and *tet*C genes, which were found in all strains. The *tet*A_2 and *tet*A_1 genes were found in 94.1% and 72.2% of isolates, respectively. In addition to the tetracycline resistance genes, all strains had lincomycin, trimethoprim, and daunorubicin resistance genes. The presence of the Tn*6188* transposon was observed in four strains, with genes encoding resistance to benzalkonium chloride, tetracyclines, and macrolides; however, just one gene (*ermC*) was found in all four strains. Interestingly just one strain isolated from smoked salmon had the *aacA4* gene, which encoded resistance to aminoglycosides. Additionally, in each of the strains, genes encoding resistance to toxic ions (*lmo1961*) were observed, including the camphor resistance protein CrcB (*lmo2082)* and aluminum (*lmo1297*). Genes encoding resistance to cadmium were observed in 47.05% (*cadC*) and 41.18% (*cadA*) of the strains, respectively (Figure 4).

### 2.5. Comparison of Isolates from Food and A Food-Production Environment with Human L. monocytogenes

One hundred and eighty-five strains of *L. monocytogenes* isolated from humans in Europe were selected for strain comparison. The results of the analysis showed that the strains causing infections most often belonged to CC6, of which 45.21% belonged to CT4915. In this study, two isolates obtained from raw salmon and fish-processing environments belong to the most frequent CT-type (CT4915).

**Figure 5:**
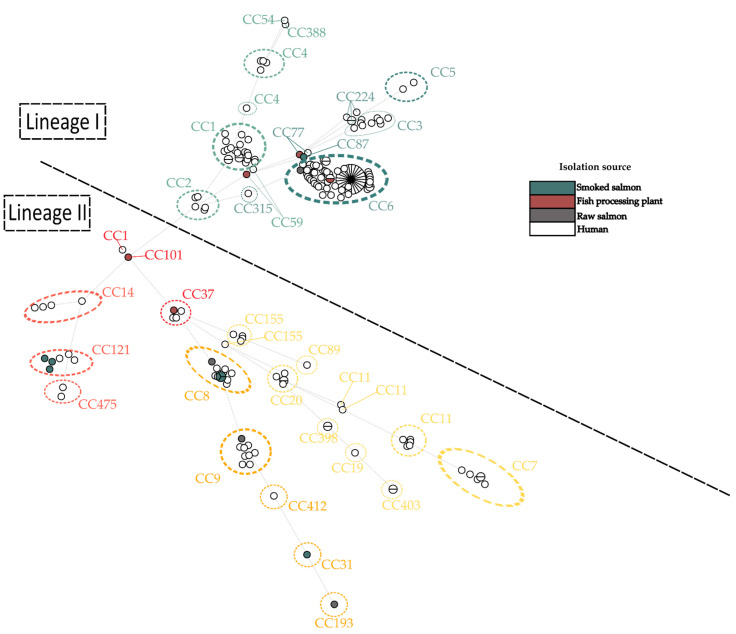
A minimum spanning tree (MST) representing the comparison of *L. monocytogenes* isolated and analyzed in this study with the published genomes recovered from listeriosis cases from Europe. Analysis was made on the base MLST profiles using a single linkage method. The graph was created with BioNumerics v7.6.

Additionally, it was noticed that four strains did not belong to any of the groups of strains isolated from humans; they were Lm_7 (CC193, SL193, and ct11716), Lm_9 (CC31, SL31, and ct7227), and Lm_17 (CC87, SL87, and ct58) (Figure 5).

**Figure 6:**
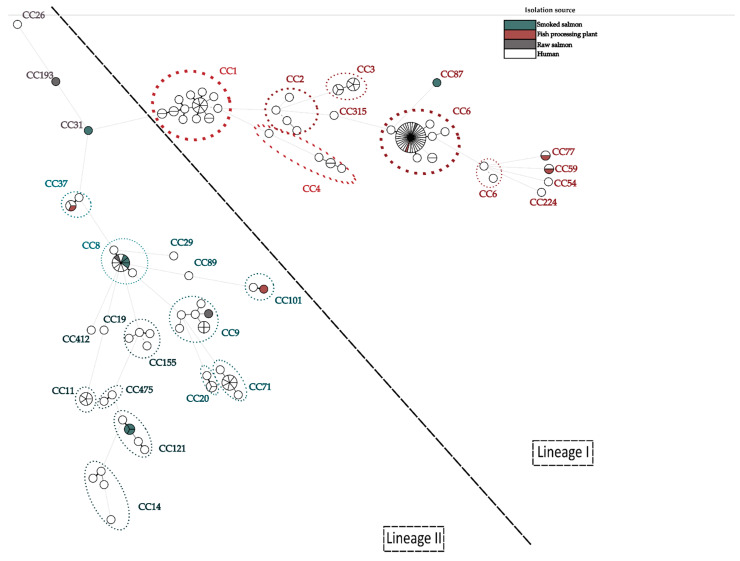
A minimum spanning tree (MST) representing the comparison of the virulence profiles of *L. monocytogenes* isolated and analyzed in this study with the virulence profile of published genomes recovered from listeriosis cases from Europe. The analysis was made on the base of MVLST profiles using a single linkage method. The graph was created with BioNumerics v7.6.

In addition, the virulence profiles were compared, which more accurately depicted the virulence potentials, and again confirmed that the Lm_7, Lm_9, and Lm_17 strains could be described as the strains with the lowest virulence potentials. The strains Lm_1 (CC8, SL8, and CT1151), Lm_2 (CC8, SL8, and CT1151), Lm_11 (CC8, SL8, and CT1151), Lm 14 (CC8, SL8, and CT7220), Lm_8 (CC6, SL6, and CT5641), and Lm_13 (CC6, SL6, and CT443) were those with the highest virulence potentials (Figure 6).

### 2.6. Detection of Prophage Regions and Plasmid

**Figure 7:**
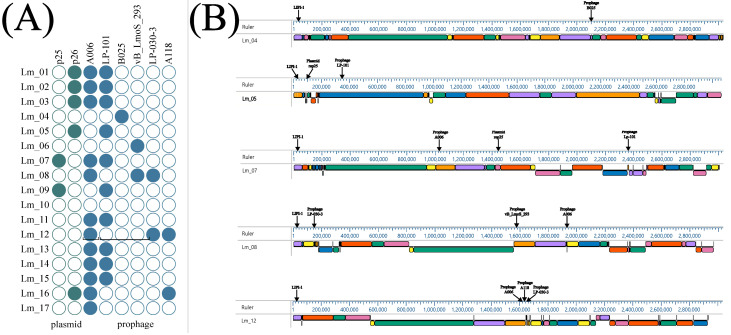
Plasmid (**A**) and prophage (**B**) profiles of isolated *L. monocytogenes* and its location in genomes.

Prophage profiles of the 17 *L. monocytogenes* genomes sequenced in this study were identified using the Prophage Hunter tool [20]. A total of six different prophage regions were found across different *L. monocytogenes* isolates (Figure 7).

The most prevalent intact prophage was the A006 [NC_009815] (*n* = 12, 64.71%), followed by the LP_101 [NC_024387] (*n* = 10, 58.82%). Three different prophages, vB LmoS _293 [NC_028929.1], LP-030-3 [NC_024384.1], and A118 [NC_003216.1], were found in 11.76% (*n* = 2). The least prevalent prophage was B025 [NC_009812.1], and it was found in just one strain: Lm_04 (CC101, SL101, and CT11711) (Figure 7A). The distribution of plasmids in the genome is shown in Figure 7B.

Analysis of the presence of plasmids revealed that in the genomes, two types of plasmids were present: pLM33 (rep25) and pLI100 (rep26). The distribution of plasmids in the genome is shown in Figure 7B.

The more common plasmid was pLI100, which was found in five strains, while the plasmid pLM33 was found in only two strains.

### 2.7. Collinearity Analysis

To investigate the genomic similarity of isolates from the same source, a collinearity relationship was constructed. The evolutionary distance among *L. monocytogenes* strains was evaluated with the DNAstar software. Each color block represented an LCB (Locally Colinear Blocks), indicating that the genome was not rearranged within this region, which was essentially distinct from genetic recombination.

Isolates from the same source were selected as a group for collinear analysis; the strains isolated from raw salmon are shown in 8A. The strains isolated from smoked salmon are shown in 8B, and the strains isolated from the fish-processing environment are shown in 8C. All genomes started with an LCB that contained LIPI-1. It was clearly seen that no strains in the isolation groups were identical.

**Figure 8:**
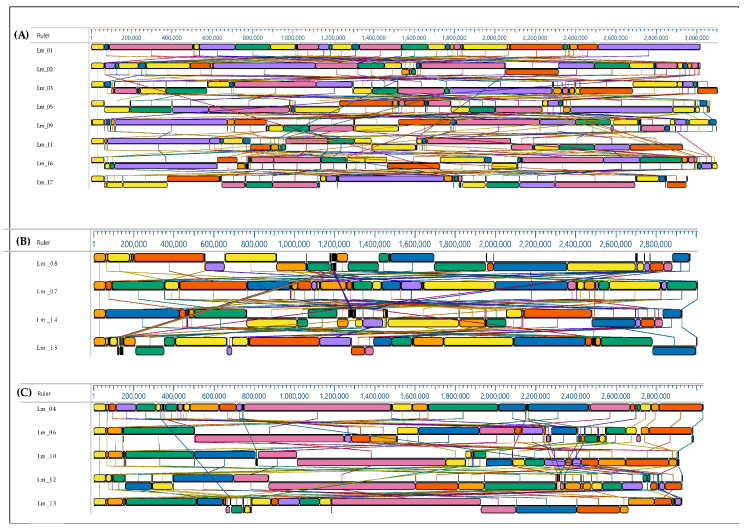
Collinearity analysis of the *L. monocytogenes* genomes isolated from smoked salmon (**A**), raw salmon (**B**) and food environment (**C**). Local collinear blocks are represented by blocks of the same color connected by lines.

The collinearity comparison chart of eight strains, Lm_01, Lm_02, Lm_03, Lm_05, Lm_09, Lm_11, Lm_16, and Lm_17, showed high heterogenicity in a group. It can clearly be seen as a translocation and inversion of LCBs (Figure 8A).

Similar conclusions can be drawn from the collinearity comparison chart of four strains: Lm_8, Lm_07, Lm_14, and Lm_15. The differences in LCBs between isolates were shown in deletions, translocation, and inversions (Figure 7B). Translocation and inversion of LCBs in genomes were observed in *L. monocytogenes* isolated from the food-processing environments (Figure 8C).

## 3. Discussion

Listeriosis is a major public health problem worldwide, and the monitoring of *L. monocytogenes* in food is mandatory and essential for risk assessment. Ready-to-eat (RTE) food of animal origin is a main source of human listeriosis. Fish products are mentioned as being frequently contaminated with *L. monocytogenes*. It should be emphasized that smoked fish, graved fish, and other fish products are most often associated with this foodborne disease [4,5,6,7,21].

Before 2000, two outbreaks were described in the Nordic countries associated with RTE from fish. The first one from Sweden concerned nine patients who consumed graved rainbow trout, and the second concerned Finland and five patients who consumed cold-smoked rainbow trout [22]. In later years, the Scandinavian countries also noted outbreaks linked to fish consumption. In 2013–15, there were three outbreaks that resulted in the deaths of seven patients and one fetus in Denmark [9], and in Sweden, there were 27 clinical cases that were due to graved and smoked fish products produced by one manufacturer [6]. The problem of listeriosis does not concern only the Nordic countries. In Germany, since 2010, there have been 22 independent outbreaks, the sources of which were smoked and/or graved salmon products [4].

In our study, it was determined that the analyzed strains belonged to eleven different MLST sequence types (ST) and fourteen different cgMLST types (CT) (Table S1). The most frequently isolated MLST types were ST8 and ST101, while the most frequently isolated cgMLST types were CT1151 and CT909. The results of other authors suggest that the dominant STs in food are ST155 and ST121 among *L. monocytogenes* of food origin [23,24,25]. It seems that such isolates may have a molecular background that allows them to survive in food and food production areas [26,27]. There are few reports characterizing *L. monocytogenes* in fish and in the fish-processing environment. Thomannsen et al. [28], analyzing the results of *L. monocytogenes* from the salmon-processing environment, showed that the most common were ST37 (88.9%) and ST8 (11.1%). Wieczorek et al. [29] characterized a total of 28 strains isolated from raw salmon, smoked salmon, and production plants that indicated that the dominant ST was ST121 (46.43%), whereas the remaining isolates belonged to ST8 (14.28%), ST155, and ST173 (10.71% each); ST31 (7.14%); and ST7 and ST504 (3.57% each). In a large study by Maury et al. [30], in which more than 400 strains isolated from seafood were characterized, it was found that over 50.0% of the strains belonged to ST121, which agrees with the results of Wieczorek et al., 2020. In our study, the number of strains belonging to ST212 was < 20%, but this can be explained by a small number of strains.

The results of our research showed a large diversity of *L. monocytogenes* strains in fish and the fish-processing environment. However, there was no straightforward evidence about the main reason for the high diversity of *L. monocytogenes* in fish. The quality of water in the place of breeding and, above all, the proximity of the agricultural runoff, have high influences on that diversity [30]. Changes in the seasons of sampling, handling, and analytical methods are likely to contribute significantly to this as well [30].

Whole genome sequencing seems to be an ideal tool for determining the virulence potentials of strains. It is an effective method for determining highly related isolates, but it can also be used to identify the presence of genes/pathogenicity islands associated with hypervirulence or modes of pathogenesis. Investigating the virulence profiles, we observed that the virulence gene counts and the comparison of virulence profiles, based on the presence of genes and their alleles, differed substantially. The LIPI-1, a Prf-A dependent virulence gene cluster, typical and crucial in the process of causing human listeriosis, was found in all the analyzed strains. In a recent study conducted by Rahman et al., three vital pathogenic proteins of L. monocytogenes, such as listeriolysin O (LLO), phosphatidylinositol-specific phospholipase C (PI-PLC), and actin polymerization protein (ActA) encoded by genes located on LIPI-1, were selected using a subtractive proteomics approach to design the multi-epitope vaccine (MEV). The authors’ in silico study showed that MEV exhibited a robust binding interaction with toll-like receptor 2 (TLR2), a key player in the innate immune system. The current subtractive proteomics and immunoinformatics study provides a background for the development of a suitable, safe, and effective vaccine against pathogenic *L. monocytogenes* [31].

Complete LIPI-2, which encodes a number of internalins and the enzyme sphingomyelinase, specific for another pathogenic species, *L. ivanovii* [32], was not found in any of the strains. Over 70% of strains possessed the *inl*I gene, which belongs to the internalin gene family; however, there was no evidence that the *inl*I gene increased the virulence potential. In fact, in a study of hypervirulent strains of *L. monocytogenes* responsible for a listeriosis outbreak in China, a unique composition of wall teichoic acids was found. Their genetic origins were in lineage I but also in the *smc*L gene located at the LIPI-2 locus [33].

The LIPI-3, which encodes a potential hemolytic factor with homology to streptolysin S (SLS), was strongly associated with lineage I strains [34]; however, the three strains found with it belonged to lineage II. The presence of the last LIPI-4 island was found in only one Lm_03 strain (lineage II, CC121, SL121, and CT909). Both the islands of LIPI-3 and LIPI-4 were associated with the hypervirulent strains responsible for an outbreak of listeriosis in Italy. The strains belonging to CC1 and CC4 had LIPI-4 present only in CC4 [35]. It was suggested that these additional pathogenicity islands are not common among food isolates and do not correlate to increased hypervirulence [36]. However, in this study, even with a small number of strains from fish, it was possible to find strains with those islands, which may also be the cause of frequent cases of listeriosis after eating fish and fish products.

The data showed the presence of PMSC in three isolates belonging to serotype IIa but of a different clonal complex (CC) and sequence type (ST). The LM_5 isolate (CC121, ST121) had a type 6 mutation (allele 49). The mutation shortened the InlA protein to a length of 491 (aa), as described before by Olier et al. [37]. The LM_7 isolate (CC193, ST193) had a type 25 mutation (allele 41). The mutation shortened the InlA protein to a length of 25 (aa), as also referred to by Moura et al. [25]. The LM_9 isolate (CC31, ST31) had a type 5 mutation (allele 40 [38]). The mutation shortened the InlA protein to a length of 188 (aa), as was referred to by Olier et al. [37].

So far, 32 types of PMSC have been detected in the *inlA* gene (Table S2). It has not been verified for all types of mutations, but it was already shown that in some isolates, the truncated *inlA* gene affected the attenuation of *L. monocytogenes* isolates and reduced their virulence.

Recent studies in South America, Europe, and Asia on the antimicrobial resistance of *L. monocytogenes* have typically reported low levels of antimicrobial resistance in isolates from food-production environments [39,40]. The latest studies have reported various antibiotic-resistance genes. Interestingly, research on WGSs of *L. monocytogenes* suggest that the dominant genes were *fosX*, *lin*, *mprF*, *norB*, and *mgrA* [41,42]; however, we found a larger group of antibiotic resistance genes. Mafuna et al. [41] reported tetracycline resistance genes, *tetM* and *tetS,* found in a few isolates, although in this study, both of those genes were not found. In strains isolated from fish and food-processing environments, the dominant genes coding tetracycline resistance genes were *tetA* and *tetC*. Tetracycline is believed to be the most frequent resistance trait in *L. monocytogenes* isolated from human and food-processing environments (REF). It is important to highlight the fact that tetracyclines averaged 20% of antibiotic classes used in aquaculture, which can result in increasing resistance against this pharmaceutic [43].

The detection of the *accA4* gene in isolate LM9 (molecular serogroup IIa (CC31, ST31)) is very interesting. In literature, the presence of this gene is associated with mobile genetic elements, such as transposons and prophages. Gene *accA4* was detected in various species, including *K. pneumoniae, P. mirabilis, P. aeruginosa, S. enterica, K. oxytoca*, *S. maltophilia, and E. cloacae* [44]. In Poland, this gene was recorded in *P. aeruginosa* isolates obtained from patients from intensive therapy units [45]. The presence of the *acca4* gene was also demonstrated by Kurpas et al. on *L. monocytogenes* isolates belonging to the molecular serotype IIb (CC5, ST5) (from the meat-production environment) [42].

In our study, after determining the presence of plasmids, it was found that 41.18% of the strains contained at least one plasmid. *L. monocytogenes* strains are known to carry plasmids with a frequency of up to 79% [46]. This information applies to clinical strains, while for isolates from food or food production, these values are lower (35%) [47]. Plasmid pLM33 is commonly found in food-related lineage II *L. monocytogenes* strains [48], but in our study, plasmid pLI100 (rep26) was more common. Due to the fact that plasmids obtained from *L. monocytogenes* have genes coding resistance to antiseptics and heavy metals, as well as chloramphenicol, clindamycin, erythromycin, streptomycin, and tetracycline [47,49,50], this increases the virulence potential and poses a potential threat for the consumers.

## 4. Materials and Methods

### 4.1. Bacterial Strains

A total of 17 *L. monocytogenes* isolate species classified by Vitek MS (bioMerieux, Marcy-l’Étoile, France) were selected for the whole genome sequencing and genomic analyses. The strains came from a collection of the Department of Industrial and Food Microbiology at University of Warmia and Mazury in Olsztyn. Strains were isolated from food samples and food-processing premises (samples were taken from both non-food contact surfaces (drains, floors, freezers, aprons, door handles, and taps) and food contact surfaces) between 2018–2019 using the standard ISO 11290-1:2017-. The identification of the species level was conducted on MALDI-TOF MS (Vitek MS, biomerieux, France) and double confirmed by performing the standard PCR proposed by Ryu at al. [51]. The strains and sources of isolation are listed in Table 1.

### 4.2. Library Preparation and Sequencing

Samples were sequenced in an external company Genomed (Warsaw, Poland). The concentration of genomic DNA was measured using the fluorimetric method using PicoGreen reagent (Life Technologies, Eugene, OR, USA). The measurement was performed on the Tecan Infinite apparatus. Genomic DNA was fragmented by sonication using Covaris E210 (Covaris, Brighton, UK). DNA Library was prepared by the NEBNext^®^ Ultra ™ II DNA Library Prep Kit for Illumina^®^ (New England Biolabs, Ipswich, MA, USA). Sequencing was made on MiSeq Illumina^®^ in PE 2 × 300 cycles with MiSeq Reagent Kit v3 reagents (600 cycles) (MS-102-3003). Readings from MiSeq were filtered with Cutadapt version 3.0. Quality control of the results was performed using the FastQC program. Denovo folding was performed by Spades version 3.14.1.

### 4.3. MLST and cgMLST Characterization

The MLST and cgMLST analyses were based on a comparison of allele profiles for 7 and 1748 genes, respectively. All calculations and determinations of allele numbers, sequence types (ST), and clonal complexes (CC) were performed by the tools available on the BIGSdb-Lm platform (https://bigsdb.pasteur.fr/listeria/ accessed on 28 September 2022). (MLST—10.1371/journal.ppat.1000146; cgMLST—10.1038/nmicrobiol.2016.185; 10.1186/1471-2105-11-595). MLST and cgMLST profile comparisons were performed using Bionumerics 7.6 software (Applied Maths, Belgium) with a single linkage algorithm.

### 4.4. Identification of Virulence-, Antimicrobial-, and Stress-Related Genes

Identification of all genes related to virulence and resistance to antimicrobials or stress factors were performed by the tools available on the BIGSdb-Lm platform (https://bigsdb.pasteur.fr/listeria/ accessed on 10 October 2022). Additionally, MVLST analysis was performed based on 93 virulence-related genes. For comparison, the strains available in the database (BIGSdb-Lm platform) were selected, and the criteria were the strain origin (Europe) and the isolation source (human). One hundred eighty-five strains of *L. monocytogenes* isolated from humans in Europe were selected.

### 4.5. Prophage and Plasmids Identification

The prophage identification was investigated using the Prophage Hunter Tool [20], used for putative prophage identification and annotation in all *L. monocytogenes* genomes. For the purpose of our analysis, only active (according to Phage Hunter) prophages were considered. Additionally, FASTA files of sequences from each strain were analyzed using PlasmidFinder 2.1 to identify predicted plasmids [52].

### 4.6. Collinearity Analysis

The evolutionary distance among *L. monocytogenes* isolates was evaluated with the DNAstar software (SeqMan NGen^®^. Version 17.2. DNASTAR. Madison, WI, USA).

## 5. Conclusions

The results indicated that *L. monocytogenes* strains isolated from fish and fish-processing premises contain a wide variety of genes encoding virulence and resistance to antimicrobials and are closely related to the CT4915 (CC6) strains that caused listeriosis in Europe. Fish and fish-processing premise isolates had high tetracycline resistance, encoded by *lmo0839*, *tet*A_3, and *tet*C genes, which were found in all strains. These strains can also cause a serious threat because many of them carry both transposons and plasmids; they can be transferred to other pathogens or commensal bacteria. The data might be useful for epidemiological investigations related to listeriosis that require more genetic information on *L. monocytogenes* from different countries in order to identify the origin of the bacterium and track these microbes along the food chain.

Future Work

Due to the enlargement of the database of genomes of L. monocytogenes isolated from fish and the fish-processing environment, in the near future, Synthetic Microbial Communities (SynCom) can be used to study the interactions between microbes and the fish matrix. The SynCom approach is an emerging research area that involves a synthetic biology approach coupled with knowledge gained from microbial community analysis and metagenomic and bioinformatics approaches (WGS). Understanding the dynamic interactions within microbial ecosystems is useful for constructing microbial consortia with robust, stable, and predictable behavior [53,54]. The holistic approaches can aim to study the fish microbiome as a whole, focusing on diminishing interferences to reduce environmental variation and elucidate how it operates in its natural environment. Additionally, SynCom can also predict microbes–host interactions; thus, it would be interesting to predict *L. monocytogenes* isolated from fish during listeriosis outbreaks.

## Figures and Tables

**Table 1:** List of *Listeria monocytogenes* strains used in study.

No.	Source	Year of Isolation	Clonal Complex	Sublineage	cgMLST Type	Serogroup
Lm_1	smoked salmon	2018	CC8	SL8	CT1151	IIa
Lm_2	smoked salmon	2018	CC8	SL8	CT1151	IIa
Lm_3	smoked salmon	2018	CC121	SL121	CT909	IIa
Lm_4	food processing premises	2018	CC101	SL101	CT11711	IIa
Lm_5	smoked salmon	2018	CC121	SL121	CT909	IIa
Lm_6	food processing premises	2018	CC59	SL59	CT2283	IIb
Lm_7	raw salmon	2018	CC193	SL193	CT11716	IIa
Lm_8	raw salmon	2018	CC6	SL6	CT5641	IVb
Lm_9	smoked salmon	2018	CC31	SL31	CT7227	IIa
Lm_10	food processing premises	2018	CC77	SL77	CT11718	IIb
Lm_11	smoked salmon	2018	CC8	SL8	CT1151	IIa
Lm_12	food processing premises	2018	CC37	SL37	CT11717	IIa
Lm_13	food processing premises	2018	CC6	SL6	CT443	IVb
Lm_14	raw salmon	2018	CC8	SL8	CT7220	IIa
Lm_15	raw salmon	2018	CC9	SL9	CT1824	IIc
Lm_16	smoked salmon	2018	CC121	SL121	CT893	IIa
Lm_17	smoked salmon	2018	CC87	SL87	CT58	IIb

## Data Availability

All genome sequences were deposited at NCBI; the corresponding BioProject Accession Number is PRJNA860799.

## Supplementary Materials

The following supporting information can be downloaded at: https://www.mdpi.com/article/10.3390/ijms24043581/s1. References [25,38,42,55,56,57,58,59,60,61,62] are cited in the Supplementary Materials.
