# Supplementary material for: A Comprehensive Virulence and Resistance Characteristics of Listeria monocytogenes Isolated from Fish and the Fish Industry Environment
Source: Int J Mol Sci. 2023 Feb 10;24(4):3581. doi: 10.3390/ijms24043581 (PMC9967382; doi:10.3390/ijms24043581)
Supplement: Supplementary file 1 [file ijms-24-03581-s001.zip › Table S2 Reported frameshifts and mutations leading to premature stop codons (PMSC) in gene inlA.pdf]

For questions and/or new additions, please contact amoura@pasteur.fr

Table S2. Reported frameshifts and mutations leading to premature stop codons (PMSC) in gene *inlA* .

Allele (BIGSdb)	PMSC mutation type[1]	Mutation position (nt)	Length truncated InlA (aa)	Lineage	Sublineage	Reference
inlA_138	**1**	1818 (T → A)	605	I	SL5	Nightingale *et al.*, 2005
inlA_125	**2**	1966 (C → T)	655	I	SL224	Nightingale *et al.*, 2005
inlA_91	**3**	2100 (C → G)	699	II	SL321	Nightingale *et al.*, 2005
inlA_28; inlA_48	**4**	12 (deletion A)	8	II	SL9, SL199	Felicio *et al.*, 2007
inlA_40	**5**	565 (C → T)	188	II	SL31	Van Stelten & Nightingale, 2008
inlA_49	**6**	1474 (C → T)	491	II	SL121	Olier *et al.*, 2003
(none yet)	**7**	1684 (C→T)	561			Van Stelten & Nightingale, 2008
inlA_43	**8**	1380 (G → A)	459	II	SL9	Rousseaux *et al.*, 2004
(none yet)	**9**	1540 (deletion G)	518			Rousseaux *et al.*, 2004
(none yet)	**10**	1961 (insertion T)	676			Rousseaux *et al.*, 2004
inlA_44; inlA_301	**11**	2054 (G → A)	684	II	SL9	Rousseaux *et al.*, 2004
(none yet)	**12**	1637 (deletion A)	576			Jonquières *et al.* , 1998
(none yet)	**13**	1579 (A → T)	526			Handa-Miya *et al.* , 2007
inlA_45	**14**	1615 (C → T)	538	II	SL9	Ragon *et al.*, 2008
inlA_35	**15**	229 (C → T)	76	II	SL13	Van Stelten *et al.*, 2010
inlA_67; inlA_69	**19**	976 (G → T)	325	II	SL9	Gelbíčová *et al.*, 2015
inlA_167	**20**	288 (C → A)	95	I	SL1	Moura *et al* ., 2016
inlA_165	**21**	806 (T → A)	268	I	SL489	Moura *et al* ., 2016
inlA_162	**22**	1756 (C → T)	585	I	SL2	Moura *et al* ., 2016
inlA_150	**23**	1939 (A → T)	646	I	SL3	Moura *et al* ., 2016
inlA_168	**24**	13 (C → T)	4	II	SL90	Moura *et al* ., 2016
inlA_41	**25**	12 (insertion A)	25	II	SL193, SL196	Moura *et al* ., 2016
inlA_158	**26**	277 (G → T)	92	II	SL7	Moura *et al* ., 2016
inlA_68	**27**	576 (insertion T)	194	II	SL9	Moura *et al* ., 2016
inlA_152	**28**	736-738 (CCA→ TAG)	245	II	SL101	Moura *et al* ., 2016
inlA_47	**29**	1635 (deletion A)	576	II	SL9	Moura *et al* ., 2016
inlA_42	**30**	1741 (C → T)	580	II	SL7	Moura *et al* ., 2016
inlA_302	**31**	2208 (deletion A)	753	I	SL5	Kurpas *et al.* , 2020
inlA_323	**32**	1041 (C → A)	346	I	SL3	Tsai *et al.* , unpublished

[1] As summarized by Gelbíčová *et al.* , 2015 and Moura *et al.* , 2016.

References

Felicio *et al.* (2007) Recurrent and sporadic *Listeria monocytogenes* contamination in alheiras represents considerable diversity, including virulence-attenuated isolates. *Appl Environ Microbiol* 73:3887–3895.

Gelbíčová *et al.* (2015) A novel mutation leading to a premature stop codon in *inlA* of *Listeria monocytogenes* isolated from neonatal listeriosis. *New Microbiol* 38:293–296.

Handa-Miya *et al.* (2007) Nonsense-mutated *inlA* and *prfA* not widely distributed in *Listeria monocytogenes* isolates from ready-to-eat seafood products in Japan. *Int J Food Microbiol* 117:312-318.

Jonquières *et al.* (1998) The *inlA* gene of *Listeria monocytogenes* LO28 harbors a nonsense mutation resulting in release of internalin. *Infect Immun* 66:3420-3422.

Kurpas M et al. (2020) Genomic characterization of Listeria monocytogenes isolated from ready-to-eat meat and meat processing environments in Poland. Front Microbiol. 11:1412.

Moura *et al.* (2016) Whole genome-based population biology and epidemiological surveillance of *Listeria monocytogenes*. *Nature Microbiol* 2:16185.

Nightingale *et al.* (2005) Select Listeria monocytogenes subtypes commonly found in foods carry disctinct nonsense mutations in inlA. Appl Environ Microbiol 71:8764–8772.

Olier *et al.* (2003) Expression of truncated internalin A is involved in impaired internalization of some *Listeria monocytogenes* isolates carried asymptomatically by humans. *Infect Immun* 71:1217–1224.

Ragon *et al.* (2008) A new perspective on *Listeria monocytogenes* evolution. *PLoS Pathog* 4.

Rousseaux *et al.* (2004) Use of PCR-restriction fragment length polymorphism of *inlA* for rapid screening of *Listeria monocytogenes* strains deficient in the ability to invade Caco-2 cells. *Appl Environ Microbiol* 70:2180–2185.

Van Stelten & Nightingale (2008) Development and implementation of a multiplex single-nucleotide polymorphism genotyping assay for detection of virulence-attenuating mutations in the *Listeria monocytogenes* virulence-associated gene *inlA. Appl Environ Microbiol* 74:7365–7375.

Van Stelten *et al.* (2010) Revelation by single-nucleotide polymorphism genotyping that mutations leading to a premature stop codon in *inlA* are common among Listeria monocytogenes isolates from ready-to-eat foods but not human listeriosis cases. *Appl Environ Microbiol* 76:2783–2790.
